# Non-High-Density Lipoprotein Cholesterol and Cardiovascular Outcomes in Chronic Kidney Disease: Results from KNOW-CKD Study

**DOI:** 10.3390/nu14183792

**Published:** 2022-09-14

**Authors:** Sang Heon Suh, Tae Ryom Oh, Hong Sang Choi, Chang Seong Kim, Eun Hui Bae, Seong Kwon Ma, Kook-Hwan Oh, Seung Hyeok Han, Soo Wan Kim

**Affiliations:** 1 Department of Internal Medicine, Chonnam National University Medical School and Chonnam National University Hospital, Gwangju 64169, Korea; 2 Department of Internal Medicine, Seoul National University Hospital, Seoul 03080, Korea; 3 Department of Internal Medicine, Institute of Kidney Disease Research, College of Medicine, Yonsei University, Seoul 03722, Korea

**Keywords:** cardiovascular events, cardiovascular risk, chronic kidney disease, mortality, non-high-density lipoprotein cholesterol

## Abstract

As non-high-density lipoprotein cholesterol (non-HDL-C) levels account for all atherogenic lipoproteins, serum non-HDL-C level has been suggested to be a marker for cardiovascular (CV) risk stratification. Therefore, to unveil the association of serum non-HDL-C levels with CV outcomes in patients with non-dialysis chronic kidney disease (ND-CKD), the patients at stages 1 to 5 (*n* = 2152) from the Korean Cohort Study for Outcomes in Patients with Chronic Kidney Disease (KNOW-CKD) were prospectively analyzed. The subjects were divided into quintiles by serum non-HDL-C level. The primary outcome was a composite of all-cause death or non-fatal CV events. The median duration of follow-up was 6.940 years. The analysis using the Cox proportional hazard model unveiled that the composite CV event was significantly increased in the 5th quintile (adjusted hazard ratio 2.162, 95% confidence interval 1.174 to 3.981), compared to that of the 3rd quintile. A fully adjusted cubic spline model depicted a non-linear, J-shaped association between non-HDL-C and the risk of a composite CV event. The association remained robust in a series of sensitivity analyses, including the analysis of a cause-specific hazard model. Subgroup analyses reveled that the association is not significantly altered by clinical conditions, including age, gender, body mass index, estimated glomerular filtration rate, and albuminuria. In conclusion, high serum non-HDL-C level increased the risk of adverse CV outcomes among the patients with ND-CKD. Further studies are warranted to define the optimal target range of non-HDL-C levels in this population.

## 1. Introduction

Deaths in chronic kidney disease (CKD) most frequently results from cardiovascular (CV) disease [1,2], while the CV risk factors, including diabetes mellitus (DM), hypertension (HTN), and dyslipidemia, are prevalent in patients with CKD [3]. As even mild impairment in the kidney function increases the risk of an adverse CV event [4], CKD is also one of the nontraditional risk factors for CV disease [5,6]. Hence, the management of CV risk factors is an issue of clinical importance for better outcomes in patients with CKD.

A decrease in high-density lipoprotein cholesterol (HDL-C) levels, along with the elevation of serum triglycerides (TG) levels, is a characteristic feature of dyslipidemia in CKD [7,8,9]. HDL-C is known to provide cardioprotection, as it is involved in reverse cholesterol transport, where excess cholesterol in peripheral tissues is cleared [10,11]. Accordingly, an inverse association between HDL-C levels and the risk of adverse CV events has been proposed [12,13,14], although a large population-based study reported a non-linear, U-shaped association between HDL-C levels and all-cause mortality [15], suggesting that the role of HDL-C as a predictor of CV outcomes may depend on various clinical circumstances [16]. In this regard, mounting evidence indicates that the antioxidant and anti-inflammatory activity of HDL-C is impaired in patients with CKD [17,18]. Another study reported that, among patients with CKD, inflammation status modifies the association of HDL-C level and the risk of adverse CV events [19]. These collectively suggest that the prediction of CV outcomes by the measurement of a single lipid parameter has only a limited value, especially in patients with CKD.

Serum non-HDL-C levels are calculated as a subtraction of HDL-C from total cholesterol [20]. As non-HDL-C accounts for all atherogenic lipoproteins, such as intermediate-density lipoprotein, lipoprotein(a), low-density lipoprotein cholesterol (LDL-C), and very LDL remnants [21], serum non-HDL-C level has been suggested to be a marker for CV risk stratification. Indeed, it has been reported that high serum non-HDL-C level is associated with an increased risk of incident CV disease in the general population and that non-HDL-C may better predict CV outcomes than conventional lipid parameters do [22,23,24,25]. However, the association between non-HDL-C and adverse CV events in patients with CKD has not yet been fully evaluated.

Therefore, we hypothesized that high serum non-HDL-C levels may predict the risk of adverse CV outcomes in patient with CKD. In the present study, we aimed to investigate the association of serum non-HDL-C levels and CV outcomes among patients with non-dialysis CKD (ND-CKD).

## 2. Materials and Methods

### 2.1. Study Design

**Figure 1:**
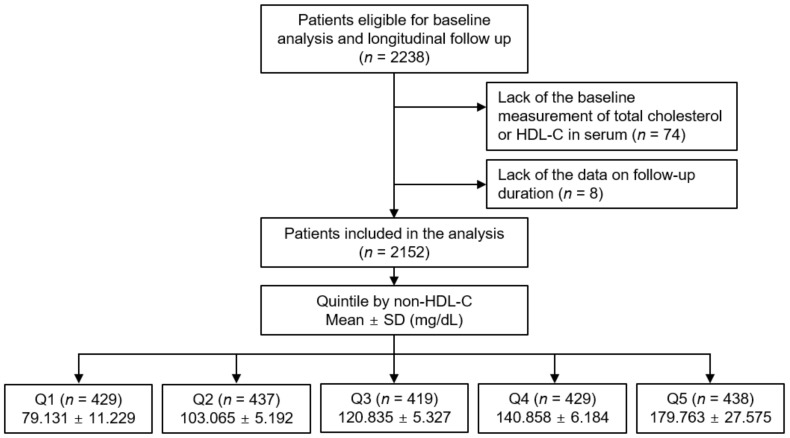
Schematic diagram of study design. Abbreviations: Non-HDL-C, non-high-density lipoprotein cholesterol; HDL-C, high-density lipoprotein cholesterol; SD, standard deviation; Q1, 1st quintile; Q2, 2nd quintile, Q3, 3rd quintile; Q4, 4th quintile; Q5, 5th quintile.

The study design of the Korean Cohort Study for Outcomes in Patients With Chronic Kidney Disease (KNOW-CKD) has been previously described (NCT01630486 at http://www.clinicaltrials.gov, accessed on 5 June 2019) [26]. Briefly, the patients with CKD at stages 1 to 5 (non-dialysis) were enrolled between 2011 and 2016. All the participants were closely monitored until 31 March 2021. Each participating center reported the study outcome events, which were cross-checked by the participating investigators. The median duration of follow-up was 6.940 years. Among those who were longitudinally followed up (*n* = 2238), after excluding those lacking the baseline measurement of total cholesterol or HDL-C in serum or those lacking the data on follow-up duration, only 2152 participants were ultimately analyzed (Figure 1). The current study followed the principles of the Declaration of Helsinki and was approved the institutional review board at each participating center (Seoul National University Hospital (1104–089-359), Seoul National University Bundang Hospital (B-1106/129–008), Yonsei University Severance Hospital (4–2011-0163), Kangbuk Samsung Medical Center (2011–01-076), Seoul St. Mary’s Hospital (KC11OIMI0441), Gil Hospital (GIRBA2553), Eulji General Hospital (201105–01), Chonnam National University Hospital (CNUH-2011-092), and Busan Paik Hospital (11–091)).

### 2.2. Data Collection from Participants

All eligible participants presented demographic information, which included age, sex, medications (statins, angiotensin-converting enzyme inhibitors and angiotensin II receptor blockers (angiotensin-converting enzyme inhibitors (ACEIs)/angiotensin receptor blockers (ARBs)), diuretic use and the number of anti-HTN drugs), Charlson comorbid index, smoking history, and primary renal disease [27]. Anthropometric measures, such as height, weight, body mass index (BMI), and systolic and diastolic blood pressures (SBP and DBP), were obtained as previously described [28]. Following overnight fasting, hemoglobin, fasting glucose, albumin, HDL-C, LDL-C, TG, total cholesterol, creatinine (Cr), 25-hydroxyvitamin D (25(OH) vitamin D), and high-sensitivity C-reactive protein (hs-CRP) levels at the baseline were determined from venous samples. Serum non-HDL-C level was defined as the subtraction of HDL-C from total cholesterol. Chronic Kidney Disease Epidemiology Collaboration equation was used to calculate the estimated glomerular filtration rate (eGFR) [29]. The classification of CKD stages followed the Kidney Disease Improving Global Outcomes guidelines [30]. Spot urine albumin-to-Cr ratio (ACR) was measured at random, preferably from second-voided urine samples. Echocardiographic data were collected from complete two-dimensional M-mode and Doppler studies following standard approaches at the participating hospitals, where the cardiologists were blinded to the clinical data. M-mode examination followed a previous guideline [31]. The echocardiographic data, such as left atrial diameter, left ventricular (LV) end diastolic diameter, the ratio of the early transmitral blood flow velocity to early diastolic velocity of the mitral annulus, valve calcification, inter-ventricular septum thickness, left ventricular (LV) ejection fraction (LVEF), regional wall motion abnormality, LV posterior wall thickness, and LV end systolic diameter were recorded [32]. The Devereux formula was used to determine LV mass [31]. LV mass index (LVMI) was determined by normalizing LV mass to height^2^ (g/m^2^).

### 2.3. Exposure and Study Outcome

The exposure of primary interest was categorized serum non-HDL-C level, where the subjects were divided into the quintile (Q1, Q2, Q3, Q4, and Q5) by serum non-HDL-C level (Figure 1). The primary outcome was composite CV event, defined as a composite of all-cause death or non-fatal CV events. Secondary outcomes included the individual outcomes of all CV events (both fatal and non-fatal), 6-point MACE, and all-cause death. The CV events included any non-fatal coronary artery events (unstable angina, myocardial infarction, or coronary revascularization or surgery), hospitalization for heart failure, cerebrovascular events (ischemic or hemorrhagic stroke, or carotid intervention), or symptomatic arrhythmia [33]. The 6-point MACE was the composite of nonfatal myocardial infarction, unstable angina, revascularization, nonfatal stroke, heart failure, symptomatic arrhythmia, or cardiac death [34]. Survival time was defined as the period between study enrollment and outcome event.

### 2.4. Statistics

The Kolmogorov–Smirnov test was used to test the normality of distribution. The baseline characteristics by serum non-HDL-C level were compared by one-way analysis of variance and χ^2^ test for continuous and categorical variates, respectively. The cumulative incidences of study outcomes were estimated by Kaplan–Meier curve analyses with log-rank test. The participants with any missing data were excluded for further analyses. Cox proportional hazard regression models were utilized to evaluate the association between serum non-HDL-C level and study outcomes, where the participants lost to follow-up were censored at the date of the last visit. Models were constructed after adjusting for the following variables. Model 1 represents crude hazard ratios (HRs). Model 2 was adjusted for age and gender. Model 3 was further adjusted for medication (statins, ACEIs/ARBs, number of anti-HTN drugs and diuretics), current smoking status, Charlson comorbidity index, primary renal disease, BMI, and SBP and DBP. Model 4 was additionally adjusted for hemoglobin, fasting glucose, albumin, hs-CRP, LDL-C, triglycerides (TG), 25(OH) vitamin D, eGFR, spot urine ACR, LVMI, and LVEF. The analysis results of Cox proportional hazard models were presented as HRs and 95% confidence intervals (CIs). The association between serum non-HDL-C levels (as a continuous variable) and HRs for study outcomes was visualized by restricted cubic splines. Our findings were validated by a series of sensitivity analyses conducted as follows. First, the subjects with CKD stage 1 were excluded, because the subjects with CKD stage 1 have nearly normal kidney function and may not be clearly affected by the burden of disease. Second, the subjects with CKD stage 5 were excluded, because the subjects with CKD stage 5 are relatively small in number, and, at the same time, the association between serum TG level and study outcomes may be exaggerated due the advanced CKD. Third, we assessed cause-specific HRs for the primary study outcome through the serum non-HDL-C level, where non-cardiac death or kidney failure with replacement therapy before the occurrence of the primary outcome were censored at the time of death and the initiation of renal replacement therapy, respectively [34]. We also conducted subgroup analyses to test whether the association of serum non-HDL-C level with study outcomes was significantly altered by clinical conditions. Subgroups were pre-specified by gender (male versus (vs.) female), age (<60 vs. ≥60 years), BMI (<23 vs. ≥23 kg/m^2^), eGFR (<45 vs. ≥45 mL/min/1.73 m^2^), and spot urine ACR (<300 vs. ≥300 mg/g) [33]. The cut-off for statistical significance was a two-sided *p* value < 0.05. Statistical analysis was performed using SPSS for Windows version 22.0 (IBM Corp., Armonk, NY, USA) and R (version 4.1.1; R project for Statistical Computing, Vienna, Austria).

## 3. Results

### 3.1. Baseline Characteristics

To describe the baseline characteristics (Table 1), the study participants were di-vided into the quintile by serum non-HDL-C level (Table 1). The mean age of the participants was higher in the subjects in Q1 than those in the Q2, Q3, Q4, and Q5. The proportion of male participants was highest in Q1. The proportion of the participants with Charlson comorbidity index 0–3 was lowest in Q1, whereas those with Charlson comorbidity index 6–7 were also most frequently observed in Q1. The history of DM was most frequent in Q1, whereas the prevalence of glomerulonephritis and PKD was highest in Q3 and Q4, respectively. The use of diuretics was most prevalent in Q5. Hemoglobin and albumin levels were lowest in Q1 and Q5, respectively. Total cholesterol, LDL-C, and TG levels were lowest in Q1, while HDL-C levels were lowest in Q5. Fasting glucose levels were highest in Q5. 25(OH) vitamin D level was significantly lower in Q5. Spot urine ACR and serum creatinine levels were significantly higher in Q5 and Q1, respectively. Accordingly, eGFR was significantly lower in Q1, while the frequency of advanced CKD was relatively higher in Q1. The echocardiographic findings of study participants by serum non-HDL-C levels did not show significant difference across the groups, except in terms of valve calcification, interventricular wall thickness, and left ventricular end-diastolic diameter (Supplementary Materials Table S1). To summarize, unfavorable clinical features were predominantly observed in Q1 and Q5.

### 3.2. Association of Serum Non-HDL-C Level with Adverse CV Events

**Figure 2:**
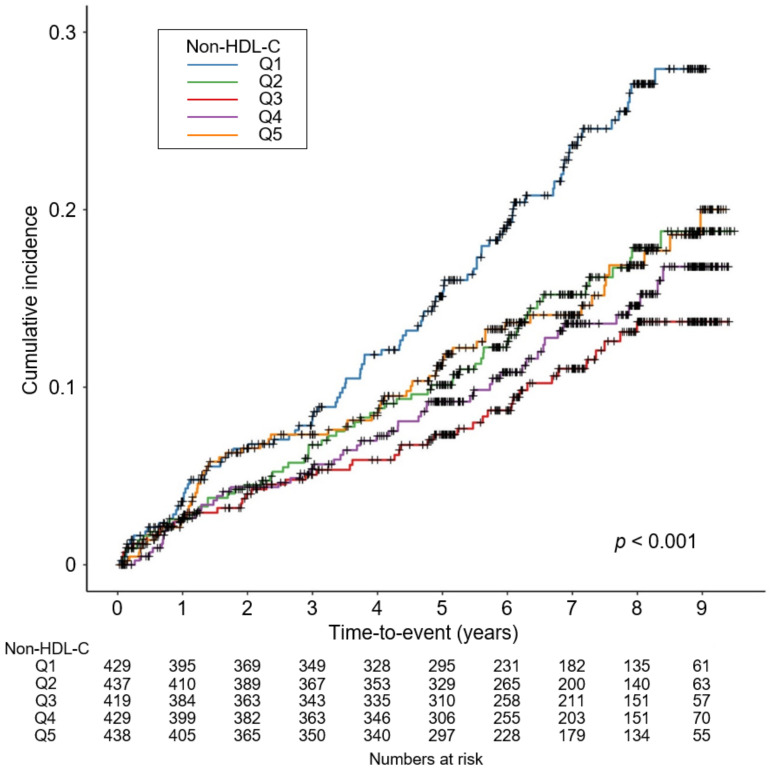
Kaplan–Meier survival curve for cumulative incidence of composite CV event by non-HDL-C. *p* value by Log-rank test. Abbreviations: CV, cardiovascular; HDL-C, high density lipoprotein cholesterol; Q1, 1st quartile; Q2, 2nd quartile; Q3, 3rd quartile; Q4, 4th quartile; Q5, 5th quintile.

**Figure 3:**
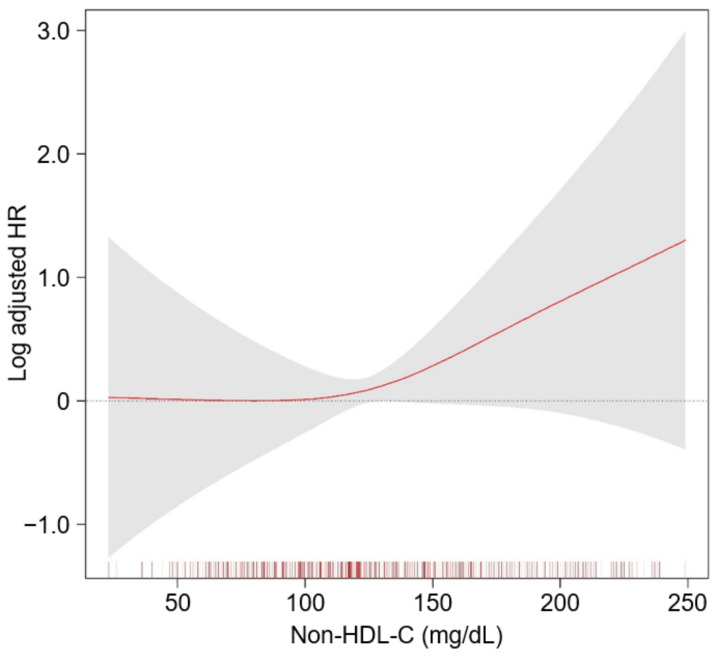
Restricted cubic spline of non-HDL-C on a composite CV event. The adjusted HR of non-HDL-C as a continuous variable for composite CV event is depicted. The model was adjusted for age and sex, Charlson comorbidity index, primary renal disease, current smoking status, medication (ACEIs/ARBs, diuretics, number of anti-HTN drugs, statins), BMI, SBP, DBP, hemoglobin, albumin, LDL-C, TG, fasting glucose, 25(OH) vitamin D, hs-CRP, eGFR, spot urine ACR, LVMI, and LVEF. Abbreviations: CI, confidence interval; HDL-C, high-density lipoprotein cholesterol; HR, hazard ratio; ACEIs, angiotensin converting enzyme inhibitors; ARBs, angiotensin receptor blocker; HTN, hypertension; BMI, body mass index; SBP, systolic blood pressure; DBP, diastolic blood pressure; LDL-C, low density lipoprotein cholesterol; TG, triglyceride; hs-CRP, high-sensitivity C-reactive protein; eGFR, estimated glomerular filtration rate; ACR, albumin-to-creatinine ratio; LVMI, left ventricular mass index; LVEF, left ventricular ejection fraction.

To determine the cumulative incidences of the primary and secondary outcomes, Kaplan–Meier curves were analyzed. The risk of a composite CV event (*p* < 0.001, by Log-rank test) was significantly differed by serum non-HDL-C level, with the lowest risk of the events in Q3 (Figure 2). The risks of all CV events (*p* = 0.003, by Log-rank test, Supplementary Materials Figure S1), 6-point MACE (*p* < 0.001, by Log-rank test, Supplementary Materials Figure S2), all-cause death (*p* = 0.015, by Log-rank test, Supplementary Materials Figure S3) were also significantly differed by serum non-HDL-C level, with the lowest risk of the events in Q3. To unveil the independent association of serum non-HDL-C level with study outcomes, Cox proportional hazard models were used. The composite CV event significantly increased in Q5 (adjusted HR 2.162, 95% CI 1.174 to 3.981), compared to that of Q3 (Table 2), suggesting that high serum non-HDL-C level increases the risk of adverse CV outcomes in patients with ND-CKD. The risks of all CV events (adjusted HR 3.350, 95% CI 1.533 to 7.321) and 6-point MACE (adjusted HR 4.298, 95% CI 1.597 to 11.569) were significantly higher in Q5, compared to that of Q3, although the risk of all-cause death was not significantly different across the groups (Table 3). Restricted cubic spline curves depicted a non-linear, J-shaped association between non-HDL-C and the risk of composite CV event (Figure 3). Similarly, restricted cubic spline curve analysis visualized the non-linear, U-shaped associations of serum non-HDL-C levels with the risk of fatal and non-fatal CV events (Supplementary Materials Figure S4), 6-point MACE (Supplementary Materials Figure S5), and all-cause death (Supplementary Materials Figure S6).

### 3.3. Sensitivity Analyses

To examine the robustness of the findings, a series of sensitivity analyses were performed. After excluding the subjects with CKD stage 1, the risk of composite CV event was significantly higher in Q4 (adjusted HR 1.737, 95% CI 1.037 to 2.910) and Q5 (adjusted HR 2.355, 95% CI 1.244 to 4.458), compared to that of Q3 (Supplementary Materials Table S2). Next, after excluding the subjects with CKD stage 5, the risk of composite CV event was still significantly higher in Q4 (adjusted HR 1.708, 95% CI 1.020 to 2.860) and Q5 (adjusted HR 2.274, 95% CI 1.202 to 4.300), compared to that of Q3 (Supplementary Materials Table S3). Finally, we analyzed cause-specific hazard model for the primary study outcome by serum non-HDL-C level, where the risk of a composite CV event remained robustly higher in Q5 (adjusted HR 2.250, 95% CI 1.178 to 4.297), compared to that of Q3 (Table 4).

### 3.4. Subgroup Analyses

To test whether the association between serum non-HDL-C level and the risk of a composite CV event is altered by certain clinical conditions, pre-specified subgroup analyses were conducted (Table 5), where the interactions between subgroups according to age, gender, BMI, eGFR, and albuminuria were tested. We could not find any significant interactions (all *p* values for interaction > 0.05), suggesting significant associations between serum non-HDL-C level with adverse CV outcomes across the aforementioned subgroups.

## 4. Discussion

In the present study, we demonstrated that high serum non-HDL-C level is associated with adverse CV outcomes in patients with ND-CKD. A fully adjusted cubic spline model depicted a non-linear, J-shaped association between non-HDL-C and the risk of CV events. The association remained robust in a series of sensitivity analyses, including the analysis of a cause-specific hazard model. Subgroup analyses reveled that the association is not significantly altered by clinical conditions, including age, gender, BMI, eGFR, and albuminuria.

Recent studies have raised a question in the traditional concept of HDL-C as a “good cholesterol”. A cohort study of the patients with CKD stage 3 to 5 (non-dialysis) reported a U-shaped association of non-HDL-C both with all-cause and CV mortality [35]. Moreover, a more dramatic paradoxical association has been reported among the patients undergoing incident hemodialysis, where all-cause and CV mortality was inversely correlated with serum HDL-C level [36]. In this context, a cohort study analyzing 1864 Korean patients with ND-CKD reported meaningful data, where inflammation status modified the association trend between serum HDL-C level and the risk of adverse CV events: Serum HDL-C level was inversely associated with the risk of CV events in the absence of inflammation, whereas the risk of CV event positively correlated with serum HDL-C level in the presence of inflammation [19]. These collectively complicate the role of HDL-C in the CV risk stratification among the patients with CKD. In the current study, we found that among the patients with CKD high serum non-HDL-C level is associated with an increased risk of adverse CV events, although the association is non-linear, suggesting that non-HDL-C may be a marker for CV risk stratification in relation to dyslipidemia.

A major finding of the current study—the association between high serum non-HDL-C level and adverse CV outcomes—is readily expectable, based on the following rationale [35]: First, the non-HDL-C level accounts for all atherogenic lipoproteins, such as intermediate-density lipoprotein, lipoprotein(a), low-density lipoprotein cholesterol (LDL-C), and LDL remnants [21]. Second, the serum non-HDL-C level is positively correlated with apolipoprotein B level, a major protein on pro-atherogenic lipoproteins [37]. Third, as LDL-C particle size inversely correlates with the serum non-HDL-C level [38], a high serum level of non-HDL-C may indicate the relative abundance of small dense LDL-C particles, which is more atherogenic.

On the other hand, it is also of note that the risk of an adverse CV event increased, but not significantly, in the subjects with very low serum non-HDL-C levels (1st and 2nd quintiles). This could be attributed to either low total cholesterol or high HDL-C levels, because non-HDL-C level is calculated by subtracting HDL-C level from total cholesterol level. The studies reporting the association of low serum total cholesterol level with an increased risk of mortality indicating a higher prevalence of malnutrition and inflammation among the subjects with low serum total cholesterol level [39,40]. It is well-known that malnutrition leads to a worsening of inflammation, accelerating the progression of atherosclerosis [41,42,43]. In addition, a high serum HDL-C level with altered anti-inflammatory property may also explain the increased risk of CV events in the subjects with very low serum non-HDL-C levels. On top of the reports indicating a decrease in the anti-inflammatory activity of HDL-C in patients with CKD [18,44,45], some reported that HDL-C could be even pro-inflammatory under uremic conditions [46,47]. Collectively, these all indicate that very low serum non-HDL-C levels may not result in favorable CV outcomes in patients with CKD.

Although LDL-C is the primary target for the management of dyslipidemia in patients with CKD, the optimal therapeutic goal in regard to serum LDL-C levels has not been specified yet [48], as statin therapy targeting serum LDL-C level in patients with CKD failed to demonstrate benefits on CV or overall survival [49]. The current Kidney Disease: Improving Global Outcomes (KDIGO) clinical practice guidelines recommend a complete evaluation of the lipid profile, including total cholesterol and HDL-C, whereas the role of serum non-HDL-C level is not determined [48]. Provided that no specific goal of dyslipidemia management in patients with CKD is established, further studies are warranted in order to define the role of serum non-HDL-C as a marker for CV risk stratification and the optimal target range of non-HDL-C level in this population.

Usui et al. [50] previously reported that high serum non-HDL-C levels are associated with an increased risk of incident coronary heart disease, where only a small portion of the subjects with CKD (357 out of 2630 subjects in total) were included. Chiu et al. [35] also reported a U-shaped association between non-HDL-C level with the risk of all-cause death and CV mortality, which also enrolled a small number of the patients with CKD (*n* = 429) and did not analyze the incidence of non-fatal CV events. In the current study, we included a total of 2521 patients with CKD at the stages 1 to 5 (non-dialysis), to present definitive evidence for the association between serum non-HDL-C level and adverse CV outcomes. A relatively long follow-up duration of up to 10 years and rigorous adjustment of potential confounders, including echocardiographic parameters (i.e., LVMI and LVEF), are additional strengths of our study.

There are a number of limitations in the present study. First, due to the observational nature of the present study, we cannot confirm the casual relation between serum non-HDL-C level and the risk of adverse CV event in patients with CKD. However, it is not difficult to provide the rationale to support the major finding of the current study [21,35,37,38]. Second, all the variables were measured once at the baseline. However, the same limitation is shared with the previous observational study, which reports similar results that are largely concordant with ours. Therefore, we assume that the limitation does not significantly interfere with the overall impact of the current study. Third, as this cohort study enrolled only ethnic Koreans, a precaution is required to extrapolate the data to other populations. It should be also noted that, however, a similar result was reported by the studies conducted in Japan and Taiwan [35,50].

In conclusion, we report that high serum non-HDL-C level is associated with adverse CV outcomes in patients with ND-CKD, suggesting that non-HDL-C may be a useful marker for CV risk stratification. Further studies are warranted to define the optimal target range of non-HDL-C levels in this population.

## Figures and Tables

**Table 1:** Baseline characteristics of study participants by non-HDL-C level.

	Non-HDL-C					
	Q1	Q2	Q3	Q4	Q5	*p* Value
Follow-up duration (year)	5.289 ± 2.790	5.399 ± 2.678	5.458 ± 2.804	5.509 ± 2.695	5.022 ± 2.874	0.087
Age (year)	56.124 ± 12.292	53.581 ± 12.387	54.084 ± 12.038	51.746 ± 11.666	53.185 ± 12.538	<0.001
Male	294 (68.5)	258 (59.0)	254 (60.6)	252 (58.7)	263 (60.0)	0.018
Charlson comorbidity index						<0.001
0–3	273 (63.6)	305 (69.8)	306 (73.0)	339 (79.0)	311 (71.0)	
4–5	146 (34.0)	123 (28.1)	109 (26.0)	83 (19.3)	122 (27.9)	
6–7	10 (2.3)	9 (2.1)	4 (1.0)	7 (1.6)	4 (0.9)	
≥8	0 (0.0)	0 (0.0)	0 (0.0)	0 (0.0)	1 (0.2)	
Primary renal disease						0.008
DM	135 (31.5)	118 (27.0)	85 (20.3)	89 (20.8)	118 (26.9)	
HTN	86 (20.0)	78 (17.8)	87 (20.8)	81 (18.9)	94 (21.5)	
GN	110 (25.6)	143 (32.7)	146 (34.8)	145 (33.9)	141 (32.2)	
TID	4 (0.9)	1 (0.2)	2 (0.5)	4 (0.9)	2 (0.5)	
PKD	57 (13.3)	72 (16.5)	70 (16.7)	81 (18.9)	60 (13.7)	
Others	37 (8.6)	25 (5.7)	29 (6.9)	28 (6.5)	23 (5.3)	
Current smoker	66 (15.4)	75 (17.2)	58 (13.8)	67 (15.7)	75 (17.2)	0.649
Medication						
ACEIs/ARBs	359 (83.7)	372 (85.1)	355 (84.7)	383 (89.3)	376 (85.8)	0.174
Diuretics	142 (33.1)	147 (33.6)	117 (27.9)	116 (27.0)	164 (37.4)	0.005
Anti-HTN drugs ≥3	131 (30.5)	125 (28.6)	113 (27.0)	124 (28.9)	137 (31.3)	0.667
Statins	319 (74.4)	266 (60.9)	209 (49.9)	141 (32.9)	183 (41.8)	<0.001
BMI (kg/m^2^)	24.160 ± 3.208	24.234 ± 3.331	24.521 ± 3.265	24.852 ± 3.493	25.248 ± 3.645	<0.001
SBP (mmHg)	125.371 ± 15.476	127.000 ± 15.409	127.150 ± 15.647	129.550 ± 16.920	129.986 ± 17.032	<0.001
DBP (mmHg)	74.028 ± 10.465	76.124 ± 10.184	77.033 ± 10.525	78.811 ± 12.113	78.542 ± 11.425	<0.001
Laboratory findings						
Hemoglobin (g/dL)	12.444 ± 1.981	12.677 ± 2.086	12.939 ± 1.930	13.198 ± 1.948	12.940 ± 2.079	<0.001
Albumin (g/dL)	4.209 ± 0.340	4.156 ± 0.370	4.217 ± 0.363	4.247 ± 0.379	4.050 ± 0.595	<0.001
Total cholesterol (mg/dL)	129.557 ± 19.156	154.018 ± 16.840	168.501 ± 14.665	190.751 ± 15.615	227.007 ± 31.829	<0.001
HDL-C (mg/dL)	50.426 ± 18.179	50.954 ± 15.841	47.666 ± 13.986	49.893 ± 14.220	47.244 ± 14.151	<0.001
LDL-C (mg/dL)	62.022 ± 13.079	80.811 ± 12.493	94.673 ± 16.827	109.538 ± 17.291	136.941 ± 29.268	<0.001
TG (mg/dL)	107.943 ± 52.589	132.076 ± 65.554	150.701 ± 73.912	171.967 ± 98.158	224.707 ± 135.411	<0.001
Fasting glucose (mg/dL)	107.498 ± 32.216	108.429 ± 39.084	111.365 ± 41.649	110.955 ± 38.180	117.199 ± 46.666	0.007
25(OH) Vitamin D (ng/mL)	18.800 ± 8.221	17.933 ± 7.500	18.426 ± 8.572	17.860 ± 8.120	16.138 ± 6.935	<0.001
hs-CRP (mg/dL)	0.600 {0.200, 1.600}	0.500 {0.200, 1.200}	0.600 {0.300, 1.700}	0.700 {0.300, 1.810}	0.900 {0.340, 2.100}	0.101
Spot urine ACR (mg/g)	282.785 {78.077, 751.305}	382.181 {83.099, 1097.469}	277.934 {44.706, 882.932}	336.845 {88.129. 938.082}	529.303 {97.796, 1908.242}	<0.001
Creatinine (mg/dL)	1.974 ± 1.272	1.856 ± 1.159	1.781 ± 1.111	1.711 ± 1.177	1.779 ± 1.033	0.022
eGFR (mL/min./1.73 m^2^)	45.966 ± 27.908	49.238 ± 30.583	50.965 ± 29.550	54.762 ± 30.663	51.207 ± 31.576	<0.001
CKD stages						0.039
Stage 1	50 (11.7)	69 (15.8)	69 (16.5)	92 (21.4)	68 (15.5)	
Stage 2	71 (16.6)	78 (17.8)	81 (19.3)	91 (21.2)	86 (19.6)	
Stage 3a	72 (16.8)	65 (14.9)	71 (16.9)	74 (17.2)	71 (16.2)	
Stage 3b	99 (23.1)	95 (21.7)	88 (21.0)	74 (17.2)	97 (22.1)	
Stage 4	106 (24.7)	97 (22.2)	89 (21.2)	70 (16.3)	94 (21.5)	
Stage 5	31 (7.2)	33 (7.6)	21 (5.0)	28 (6.5)	22 (5.0)	

Values for categorical variables are given as numbers (percentage); values for continuous variables, as mean ± standard deviation or median {interquartile range}. Abbreviations: ACEIs, angiotensin converting enzyme inhibitors; ACR, albumin-to-creatinine ratio; ARB, angiotensin receptor blocker; BMI, body mass index; CCI, Charlson comorbidity index; CKD, chronic kidney disease; DBP, diastolic blood pressure; DM, diabetes mellitus; eGFR, estimated glomerular filtration rate; GN, glomerulonephritis; HDL-C, high density lipoprotein cholesterol; hs-CRP, high-sensitivity C-reactive protein; HTN, hypertension; LDC-C, low density lipoprotein cholesterol; PKD, polycystic kidney disease; SBP, systolic blood pressure; TG, triglyceride; TID, tubulointerstitial disease; Q1, 1st quintile; Q2, 2nd quintile; Q3, 3rd quintile; Q4, 4th quintile; Q5, 5th quintile; UAGT/Cr, urinary angiotensinogen-to-creatinine ratio.

**Table 2:** HRs for the primary outcome by non-HDL-C level.

	Non-HDL-C	Events, *n* (%)	Model 1		Model 2		Model 3		Model 4	
			HR (95%CIs)	*p* Value	HR (95%CIs)	*p* Value	HR (95%CIs)	*p* Value	HR (95%CIs)	*p* Value
Composite CV event	Q1	58 (13.5)	2.208 (1.465, 3.3328)	<0.001	1.840 (1.280, 2.646)	<0.001	1.786 (1.228, 2.598)	0.002	1.408 (0.837, 2.368)	0.197
	Q2	40 (9.2)	1.548 (1.006, 2.384)	0.047	1.473 (0.998, 2.173)	0.051	1.404 (0.946, 2.084)	0.092	1.432 (0.909, 2.257)	0.121
	Q3	26 (6.2)	Reference		Reference		Reference		Reference	
	Q4	32 (7.5)	1.259 (0.800, 1.982)	0.320	1.423 (0.951, 2.131)	0.087	1.293 (0.859, 1.947)	0.217	1.512 (0.926, 2.469)	0.098
	Q5	42 (9.6)	1.558 (1.004, 2.418)	0.048	1.542 (1.042, 2.281)	0.030	1.486 (0.995, 2.219)	0.053	2.162 (1.174, 3.981)	0.013

Model 1, unadjusted model. Model 2, model 1 + adjusted for age and sex. Model 3, model 2 + adjusted Charlson comorbidity index, primary renal disease, current smoking status, medication (ACEIs/ARBs, diuretics, number of anti-HTN drugs, statins), BMI, SBP, and DBP. Model 4, model 3 + adjusted for hemoglobin, albumin, LDL-C, TG, fasting glucose, 25(OH) vitamin D, hs-CRP, eGFR, spot urine ACR, LVMI, and LVEF. Abbreviations: CI, confidence interval; HDL-C, high-density lipoprotein cholesterol; HR, hazard ratio; Q1, 1st quintile; Q2, 2nd quintile; Q3, 3rd quintile; Q4, 4th quintile; Q5, 5th quintile.

**Table 3:** HRs for the secondary outcomes by non-HDL-C level.

	Non-HDL-C	Events, *n* (%)	Model 1		Model 2		Model 3		Model 4	
			HR (95%CIs)	*p* Value	HR (95%CIs)	*p* Value	HR (95%CIs)	*p* Value	HR (95%CIs)	*p* Value
All CV events	Q1	25 (6.0)	2.167 (1.242, 3.781)	0.006	1.952 (1.216, 3.314)	0.006	1.835 (1.127, 2.990)	0.014	1.266 (0.629, 2.549)	0.509
	Q2	56 (13.1)	1.598 (0.894, 2.856)	0.114	1.449 (0.870, 2.414)	0.155	1.387 (0.827, 2.325)	0.215	1.445 (0.785, 2.660)	0.238
	Q3	36 (8.2)	Reference		Reference		Reference		Reference	
	Q4	31 (7.2)	1.235 (0.666, 2.288)	0.503	1.388 (0.818, 2.353)	0.224	1.298 (0.761, 2.215)	0.338	1.547 (0.800, 2.988)	0.195
	Q5	39 (8.9)	1.864 (1.050, 1.068)	0.034	1.684 (1.019, 2.783)	0.042	1.644 (0.984, 2.746)	0.058	3.350 (1.533, 7.321)	0.002
6-point MACE	Q1	14 (3.3)	2.906 (1.420, 5.945)	0.004	2.655 (1.451, 4.856)	0.002	2.279 (1.227, 4.233)	0.009	1.467 (0.603, 3.568)	0.398
	Q2	44 (10.3)	2.304 (1.107, 4.797)	0.026	2.021 (1.064, 3.839)	0.031	1.774 (0.928, 3.392)	0.083	1.862 (0.859, 4.034)	0.115
	Q3	28 (6.4)	Reference		Reference		Reference		Reference	
	Q4	22 (5.1)	1.559 (0.707, 3.435)	0.271	1.834 (0.937, 3.591)	0.077	1.768 (0.896, 3.489)	0.100	2.124 (0.912, 4.949)	0.081
	Q5	27 (6.2)	2.239 (1.060, 4.728)	0.035	2.073 (1.087, 3.955)	0.027	2.163 (1.035, 4.168)	0.021	4.298 (1.597, 11.569)	0.004
All-cause death	Q1	22 (5.3)	2.062 (1.210, 3.512)	0.008	1.663 (0.999, 2.766)	0.050	1.606 (0.947, 2.723)	0.079	1.669 (0.828, 3.363)	0.152
	Q2	47 (11.0)	1.515 (0.866, 2.648)	0.145	1.514 (0.883, 2.598)	0.132	1.270 (0.733, 2.203)	0.394	1.461 (0.804, 2.655)	0.213
	Q3	33 (7.6)	Reference		Reference		Reference		Reference	
	Q4	27 (6.3)	1.228 (0.682, 2.211)	0.494	1.492 (0.848, 2.627)	0.165	1.332 (0.749, 2.366)	0.329	1.467 (0.771, 2.794)	0.243
	Q5	26 (5.9)	1.105 (0.603, 2.025)	0.747	1.253 (0.710, 2.211)	0.437	1.133 (0.636, 2.019)	0.672	0.891 (0.374, 2.122)	0.794

Model 1, unadjusted model. Model 2, model 1 + adjusted for age and sex. Model 3, model 2 + adjusted Charlson comorbidity index, primary renal disease, current smoking status, medication (ACEIs/ARBs, diuretics, number of anti-HTN drugs, statins), BMI, SBP, and DBP. Model 4, model 3 + adjusted for hemoglobin, albumin, LDL-C, TG, fasting glucose, 25(OH) vitamin D, hs-CRP, eGFR, spot urine ACR, LVMI, and LVEF. Abbreviations: CI, confidence interval; HDL-C, high-density lipoprotein cholesterol; HR, hazard ratio; Q1, 1st quintile; Q2, 2nd quintile; Q3, 3rd quintile; Q4, 4th quintile; Q5, 5th quintile.

**Table 4:** Cause-specific HRs for the primary outcome by non-HDL-C level.

	Non-HDL-C	Model 1		Model 2		Model 3		Model 4	
		HR (95%CIs)	*p* Value	HR (95%CIs)	*p* Value	HR (95%CIs)	*p* Value	HR (95%CIs)	*p* Value
Composite CV event	Q1	2.242 (1.563, 3.217)	<0.001	1.790 (1.242, 2.581)	0.002	1.666 (1.140, 2.434)	0.008	1.458 (0.891, 2.386)	0.134
	Q2	1.439 (0.975, 2.125)	0.067	1.517 (1.025, 2.245)	0.037	1.436 (0.970, 2.126)	0.070	1.527 (0.980, 2.377)	0.061
	Q3	Reference		Reference		Reference		Reference	
	Q4	1.120 (0.801, 1.793)	0.378	1.441 (0.960, 2.164)	0.078	1.329 (0.883, 1.999)	0.173	1.527 (0.925, 2.520)	0.098
	Q5	1.486 (1.002, 2.023)	0.048	1.537 (1.038, 2.276)	0.032	1.477 (0.934, 2.218)	0.060	2.250 (1.178, 4.297)	0.014

Model 1, unadjusted model. Model 2, model 1 + adjusted for age and sex. Model 3, model 2 + adjusted Charlson comorbidity index, primary renal disease, current smoking status, medication (ACEIs/ARBs, diuretics, number of anti-HTN drugs, statins), BMI, SBP, and DBP. Model 4, model 3 + adjusted for hemoglobin, albumin, LDL-C, TG, fasting glucose, 25(OH) vitamin D, hs-CRP, eGFR, spot urine ACR, LVMI, and LVEF. Abbreviations: CI, confidence interval; HDL-C, high-density lipoprotein cholesterol; HR, hazard ratio; Q1, 1st quintile; Q2, 2nd quintile; Q3, 3rd quintile; Q4, 4th quintile; Q5, 5th quintile.

**Table 5:** HRs for the primary outcome by non-HDL-C level in various subgroups.

	Non-HDL-C	Events, *n* (%)	Unadjusted HR (95%CIs)	*p* for Interaction	Adjusted HR (95%CIs)	*p* for Interaction
Age <60 years	Q1	32 (13.3)	2.332 (1.279, 4.250)	0.559	1.641 (0.669, 4.025)	0.486
	Q2	25 (9.1)	1.525 (0.814, 2.857)		1.662 (0.796, 3.468)	
	Q3	16 (5.9)	Reference		Reference	
	Q4	27 (8.8)	1.538 (0.828, 2.854)		1.656 (0.784, 3.498)	
	Q5	20 (6.9)	1.254 (0.650, 2.420)		1.486 (0.531, 4.158)	
Age ≥60 years	Q1	61 (32.4)	1.821 (1.157, 2.856)		1.328 (0.680, 2.594)	
	Q2	37 (23.0)	1.281 (0.780, 2.104)		1.379 (0.755, 2.517)	
	Q3	27 (18.4)	Reference		Reference	
	Q4	26 (21.3)	1.083 (0.632, 1.856)		1.458 (0.738, 2.881)	
	Q5	40 (26.8)	1.584 (0.972, 2.581)		2.568 (1.176, 5.605)	
Male	Q1	74 (25.2)	2.351 (1.530, 3.613)	0.838	1.129 (0.611, 2.088)	0.205
	Q2	43 (16.7)	1.512 (0.944, 2.422)		1.366 (0.793, 2.353)	
	Q3	29 (11.4)	Reference		Reference	
	Q4	36 (14.3)	1.248 (0.765, 2.035)		1.836 (1.012, 3.332)	
	Q5	45 (17.1)	1.637 (1.027, 2.612)		3.209 (1.563, 6.587)	
Female	Q1	19 (14.1)	1.664 (0.834, 3.319)		2.724 (0.996, 7.447)	
	Q2	19 (10.6)	1.191 (0.597, 2.376)		1.449 (0.617, 3.399)	
	Q3	14 (8.5)	Reference		Reference	
	Q4	17 (9.6)	1.116 (0.550, 2.264)		1.252 (0.502, 3.124)	
	Q5	15 (8.6)	1.045 (0.504, 2.165)		1.030 (0.305, 3.484)	
BMI <23 kg/m^2^	Q1	31 (19.9)	1.910 (1.031, 3.538)	0.206	1.613 (0.610, 4.265)	0.554
	Q2	28 (17.4)	1.545 (0.825, 2.892)		1.912 (0.865, 4.225)	
	Q3	15 (10.8)	Reference		Reference	
	Q4	9 (7.3)	0.644 (0.282, 1.472)		0.912 (0.347, 2.417)	
	Q5	15 (12.7)	1.350 (0.660, 2.762)		1.518 (0.426, 5.406)	
BMI ≥23 kg/m^2^	Q1	62 (22.9)	2.402 (1.537, 3.753)		1.389 (0.721, 2.676)	
	Q2	34 (12.6)	1.277 (0.774, 2.105)		1.248 (0.691, 2.252)	
	Q3	28 (10.1)	Reference		Reference	
	Q4	44 (14.6)	1.450 (0.903, 2.330)		1.930 (1.061, 3.508)	
	Q5	45 (14.1)	1.458 (0.909, 2.336)		2.718 (1.299, 5.684)	
eGFR ≥45 mL/min./1.73 m^2^	Q1	32 (18.1)	2.647 (1.413, 4.961)	0.601	2.688 (0.955, 7.562)	0.859
	Q2	21 (10.6)	1.503 (0.764, 2.957)		2.013 (0.869, 4.663)	
	Q3	14 (6.7)	Reference		Reference	
	Q4	21 (8.5)	1.211 (0.616, 2.381)		1.844 (0.765, 4.442)	
	Q5	17 (8.1)	1.234 (0.608, 2.504)		2.593 (0.784, 8.578)	
eGFR <45 mL/min./1.73 m^2^	Q1	61 (24.2)	1.917 (1.232, 2.984)		1.213 (0.650, 2.265)	
	Q2	41 (17.2)	1.301 (0.809, 2.094)		1.232 (0.702, 2.161)	
	Q3	29 (13.9)	Reference		Reference	
	Q4	32 (17.6)	1.334 (0.807, 2.205)		1.601 (0.870, 2.946)	
	Q5	43 (18.8)	1.518 (0.948, 2.432)		2.185 (1.033, 4.625)	
Spot urine ACR <300 mg/g	Q1	44 (20.8)	2.739 (1.565, 4.793)	0.286	2.242 (1.059, 5.227)	0.552
	Q2	28 (15.2)	1.981 (1.084, 3.620)		1.978 (0.993, 3.942)	
	Q3	17 (8.1)	Reference		Reference	
	Q4	17 (8.7)	1.055 (0.538, 2.066)		1.109 (0.783, 2.448)	
	Q5	19 (12.0)	1.565 (0.813, 3.011)		2.032 (0.783, 5.272)	
Spot urine ACR ≥300 mg/g	Q1	44 (21.7)	1.741 (1.065, 2.844)		1.043 (0.511, 2.130)	
	Q2	33 (14.2)	1.075 (0.639, 1.808)		1.146 (0.617, 2.126)	
	Q3	25 (12.8)	Reference		Reference	
	Q4	34 (15.4)	1.199 (0.715, 2.009)		1.723 (0.911, 3.258)	
	Q5	40 (15.1)	1.259 (0.764, 2.076)		2.147 (0.943, 4.889)	

Adjusted HR of non-HDL-C as a continuous variable for all-cause death is depicted. The model was adjusted for age and sex, Charlson comorbidity index, primary renal disease, current smoking status, medication (ACEIs/ARBs, diuretics, number of anti-HTN drugs, statins), BMI, SBP, DBP, hemoglobin, albumin, LDL-C, TG, fasting glucose, 25(OH) vitamin D, hs-CRP, eGFR, spot urine ACR, LVMI, and LVEF. Abbreviations: CI, confidence interval; eGFR, estimated glomerular filtration rate; HDL-C, high-density lipoprotein cholesterol; HR, hazard ratio; Q1, 1st quintile; Q2, 2nd quintile; Q3, 3rd quintile; Q4, 4th quintile; Q5, 5th quintile.

## Data Availability

Not applicable.

## Supplementary Materials

The following are available online at https://www.mdpi.com/article/10.3390/nu14183792/s1, Figure S1: Kaplan-Meier survival curve for cumulative incidence of fatal and non-fatal CV event by non-HDL-C; Figure S2: Kaplan–Meier survival curve for cumulative incidence of 6-point MACE by non-HDL-C; Figure S3: Kaplan–Meier survival curve for cumulative incidence of all-cause death by non-HDL-C; Figure S4: Restricted cubic spline of non-HDL-C on fatal and non-fatal CV event; Figure S5: Restricted cubic spline of non-HDL-C on 6-point MACE; Figure S6: Restricted cubic spline of non-HDL-C on all-cause death; Table S1. Summary of echocardiographic findings of study participants by non-HDL-C; Table S2: HRs for the primary outcome by non-HDL-C level after excluding the subjects at CKD stage 1; Table S3: HRs for the primary outcome by non-HDL-C level after excluding the subjects at CKD stage 5.
