# Supplementary material for: Non-High-Density Lipoprotein Cholesterol and Cardiovascular Outcomes in Chronic Kidney Disease: Results from KNOW-CKD Study
Source: Nutrients. 2022 Sep 14;14(18):3792. doi: 10.3390/nu14183792 (PMC9505887; doi:10.3390/nu14183792)
Supplement: Supplementary file 1 [file nutrients-14-03792-s001.zip › nutrients-1929063-supplementary.pdf]

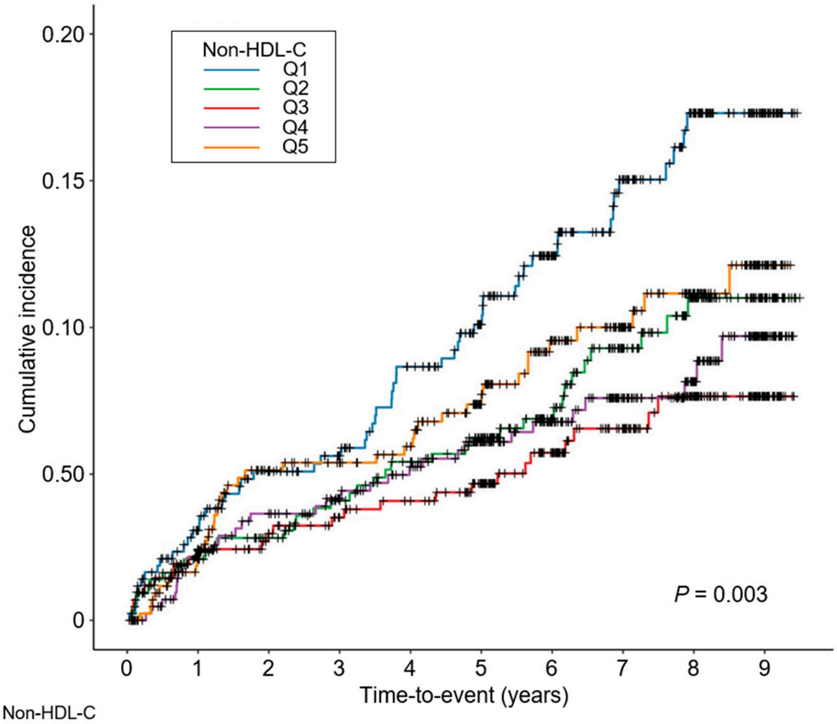

Non-HDL-C										
Q1	429	395	369	349	328	295	231	182	135	61
Q2	437	410	389	367	353	329	265	200	140	63
Q3	419	384	363	343	335	310	258	211	151	57
Q4	429	399	382	363	346	306	255	203	151	70
Q5	438	405	365	350	340	297	228	179	134	55

Numbers at risk

Figure S1. Kaplan-Meier survival curve for cumulative incidence of all CV events by non-HDL-C. Note: *P* value by Log-rank test. Abbreviations: CV, cardiovascular; HDL-C, high density lipoprotein cholesterol; Q1, 1st quintile; Q2, 2nd quintile; Q3, 3rd quintile; Q4, 4th quintile; Q5, 5th quintile.

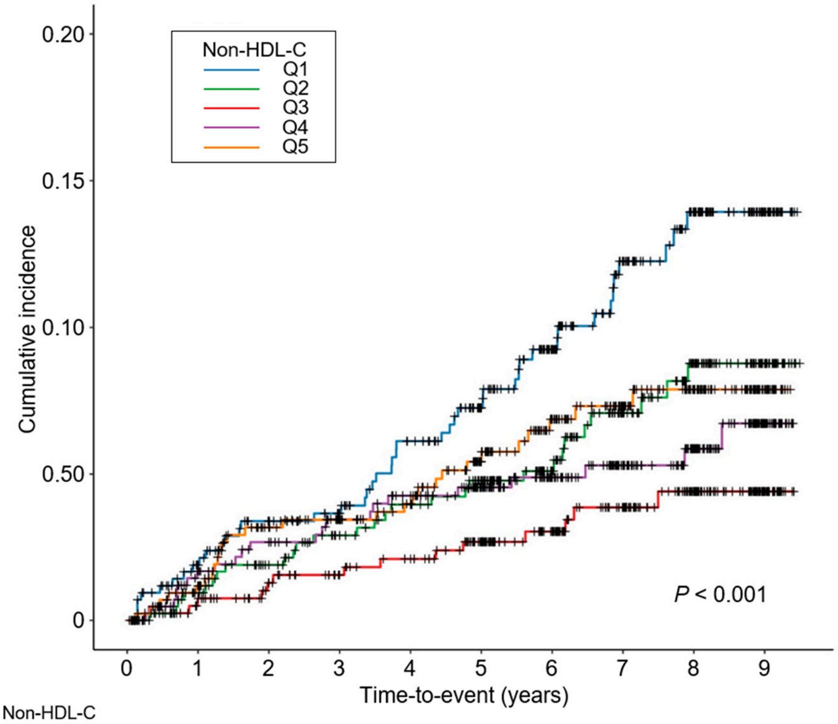

Non-HDL-C										
Q1	429	401	378	359	340	308	243	189	141	65
Q2	437	417	396	374	361	337	370	306	145	66
Q3	419	391	370	352	344	318	266	218	158	59
Q4	429	402	386	366	351	312	259	206	152	71
Q5	438	409	374	359	349	306	236	185	140	58

Numbers at risk

Figure S2. Kaplan-Meier survival curve for cumulative incidence of 6-point MACE by non-HDL-C. Note: *P* value by Log-rank test. Abbreviations: HDL-C, high density lipoprotein cholesterol; Q1, 1st quintile; Q2, 2nd quintile; Q3, 3rd quintile; Q4, 4th quintile; Q5, 5th quintile.

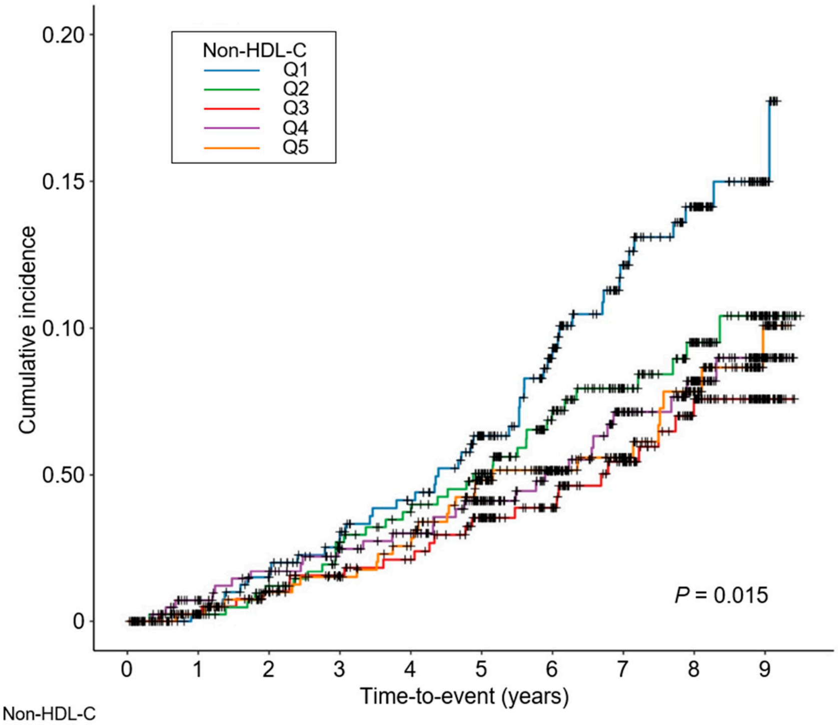

Non-HDL-C										
Q1	429	406	386	367	355	324	256	203	154	73
Q2	437	420	403	384	373	350	281	271	156	71
Q3	419	394	374	356	349	322	270	223	161	60
Q4	429	407	392	374	362	319	266	212	157	76
Q5	438	413	386	372	362	320	249	197	147	62

Numbers at risk

Figure S3. Kaplan-Meier survival curve for cumulative incidence of all-cause death by non-HDL-C. Note: P value by Log-rank test. Abbreviations: HDL-C, high density lipoprotein cholesterol; Q1, 1st quintile; Q2, 2nd quintile; Q3, 3rd quintile; Q4, 4th quintile; Q5, 5th quintile.

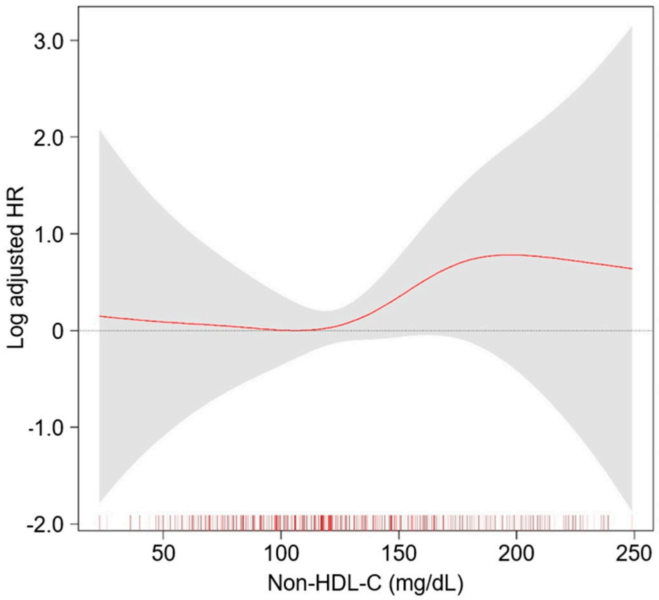

Figure S4. Restricted cubic spline of non-HDL-C on all CV events. Note: Adjusted HR of non-HDL-C as a continuous variable for fatal and non-fatal CV event is depicted. The model was adjusted for age and sex, Charlson comorbidity index, primary renal disease, current smoking status, medication (ACEIs/ARBs, diuretics, number of anti-HTN drugs, statins), BMI, SBP, DBP, hemoglobin, albumin, LDL-C, TG, fasting glucose, 25(OH) vitamin D, hs-CRP, eGFR, spot urine ACR, LVMI, and LVEF. Abbreviations: CI, confidence interval; HDL-C, high-density lipoprotein cholesterol; HR, hazard ratio; ACEIs, angiotensin-converting enzyme inhibitors; ARBs, angiotensin receptor blockers; ; HTN, hypertension; BMI, body mass index; SBP, systolic blood pressure; DBP, diastolic blood pressure; LDL-C, low-density lipoprotein cholesterol; TG, total triglycerides; hs-CRP, high-sensitivity C-reactive protein; eGFR, estimated glomerular filtration rate; ACR, albumin-to-creatinine ratio; LVMI, left ventricular mass index; LVEF, left ventricular ejection fraction; Q1, 1st quintile; Q2, 2nd quintile; Q3, 3rd quintile; Q4, 4th quintile; Q5, 5th quintile.

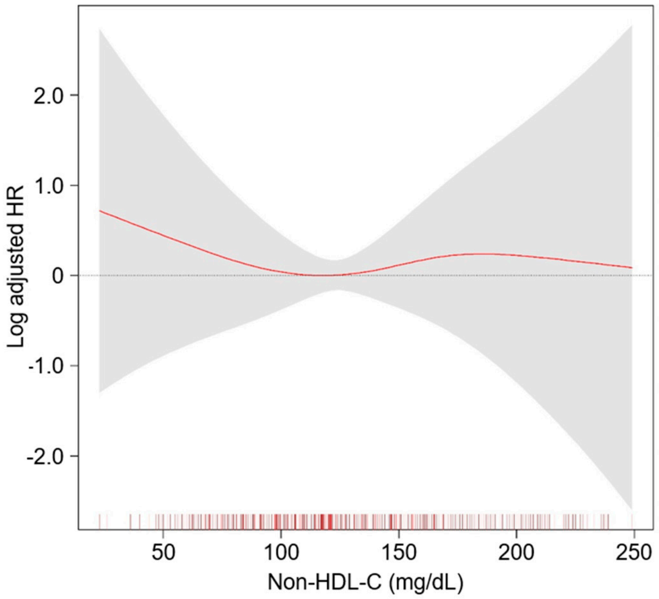

Figure S5. Restricted cubic spline of non-HDL-C on 6-point MACE. Note: Adjusted HR of non-HDL-C as a continuous variable for 6-point MACE is depicted. The model was adjusted for age and sex, Charlson comorbidity index, primary renal disease, current smoking status, medication (ACEIs/ARBs, diuretics, number of anti-HTN drugs, statins), BMI, SBP, DBP, hemoglobin, albumin, LDL-C, TG, fasting glucose, 25(OH) vitamin D, hs-CRP, eGFR, spot urine ACR, LVMI, and LVEF. Abbreviations: CI, confidence interval; HDL-C, high-density lipoprotein cholesterol; HR, hazard ratio; Q1, 1st quintile; Q2, 2nd quintile; Q3, 3rd quintile; Q4, 4th quintile; Q5, 5th quintile.

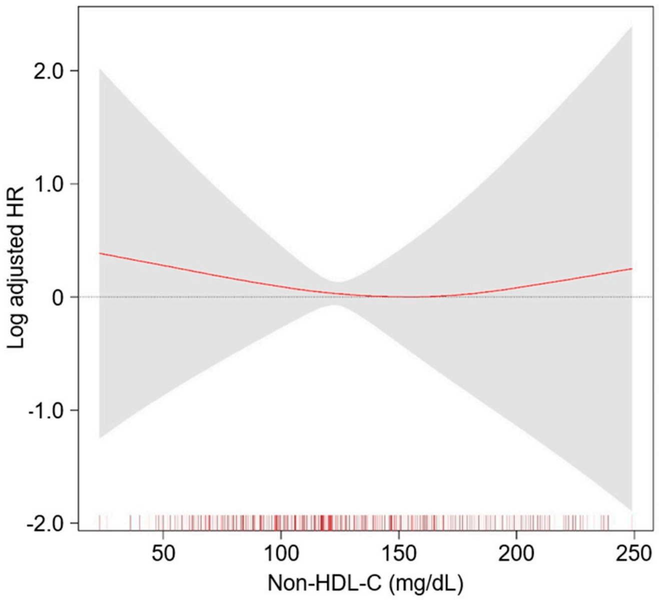

Figure S6. Restricted cubic spline of non-HDL-C on all-cause death. Note: Adjusted HR of non-HDL-C as a continuous variable for all-cause deateh is depicted. The model was adjusted for age and sex, Charlson comorbidity index, primary renal disease, current smoking status, medication (ACEIs/ARBs, diuretics, number of anti-HTN drugs, statins), BMI, SBP, DBP, hemoglobin, albumin, LDL-C, TG, fasting glucose, 25(OH) vitamin D, hs-CRP, eGFR, spot urine ACR, LVMI, and LVEF. Abbreviations: CI, confidence interval; HDL-C, high-density lipoprotein cholesterol; HR, hazard ratio; Q1, 1st quintile; Q2, 2nd quintile; Q3, 3rd quintile; Q4, 4th quintile; Q5, 5th quintile.

Table S1. Summary of echocardiographic findings of study participants by non-HDL-C.

	Non-HDL-C					
	**Q1**	**Q2**	**Q3**	**Q4**	**Q5**	***P* value**
LVMI (g/m²)	93.605 ± 24.844	94.014 ± 25.843	92.128 ± 23.836	95.431 ± 27.508	94.121 ± 24.155	0.484
E/e′	10.047 ± 3.694	9.820 ± 3.359	9.876 ± 4.502	9.937 ± 4.039	10.197 ± 3.898	0.618
LVEF (%)	63.899 ± 6.353	63.883 ± 6.042	64.328 ± 6.256	63.795 ± 6.811	64.491 ± 6.075	0.388
LAD (mm)	38.496 ± 6.286	37.879 ± 5.836	37.492 ± 5.830	37.799 ± 5.871	37.705 ± 5.497	0.176
RWMA	20 (4.7)	12 (2.8)	10 (2.4)	13 (3.1)	11 (2.6)	0.389
Valve calcification	47 (11.1)	44 (10.3)	35 (8.4)	21 (4.9)	39 (9.1)	0.018
PWT (mm)	9.274 ± 1.555	9.201 ± 1.658	9.199 ± 1.535	9.336 ± 1.636	9.388 ± 1.550	0.322
IVWT (mm)	9.283 ± 1.664	9.261 ± 1.799	9.262 ± 1.600	9.457 ± 1.823	9.610 ± 1.715	0.008
LVEDD (mm)	48.842 ± 4.717	48.938 ± 4.510	48.595 ± 4.429	48.909 ± 4.571	48.110 ± 4.293	0.032
LVESD (mm)	30.620 ± 4.289	30.481 ± 4.345	30.324 ± 4.231	30.576 ± 4.390	30.014 ± 4.131	0.218

Note: Values for categorical variables are given as number (percentage); values for continuous variables, as mean ± standard deviation or median (interquartile range). Abbreviations: E/e′, ratio of the early transmitral blood flow velocity to early diastolic velocity of the mitral annulus; HDL-C, high density lipoprotein cholesterol; IVWT, interventricular wall thickness; LAD, left atrium diameter; LVEDD, left ventricular end-diastolic diameter; LVEF, left ventricular ejection fraction; LVESD, left ventricular end-systolic diameter; LVMI, left ventricular mass index; PWT, posterior wall thickness; RMWA, regional wall motion abnormality; Q1, 1st quintile; Q2, 2nd quintile; Q3, 3rd quintile; Q4, 4th quintile; Q5, 5th quintile.

Table S2. HRs for the primary outcome by non-HDL-C level after excluding the subjects at CKD stage 1.

	Non-HDL-C	Events, *n* (%)	Model 1		Model 2		Model 3		Model 4	
			HR (95%CIs)	*P* value	HR (95%CIs)	*P* value	HR (95%CIs)	*P* value	HR (95%CIs)	*P* value
Composite CV event	Q1	93 (24.5)	2.445 (1.591, 3.758)	<0.001	2.024 (1.390, 2.946)	<0.001	1.937 (1.314, 2.855)	<0.001	1.598 (0.931, 2.744)	0.089
	Q2	60 (16.3)	1.723 (1.095, 2.710)	0.019	1.569 (1.048, 2.349)	0.029	1.488 (0.988, 2.241)		1.573 (0.976, 2.535)	0.063
	Q3	39 (11.1)	Reference		Reference		Reference		Reference	
	Q4	48 (14.2)	1.441 (0.893, 2.324)	0.134	1.500 (0.982, 2.291)	0.061	1.364 (0.888, 2.095)	0.156	1.737 (1.037, 2.910)	0.036
	Q5	57 (15.4)	1.693 (1.066, 2.690)	0.026	1.623 (1.079, 2.441)	0.020	1.565 (1.032, 2.375)	0.035	2.355 (1.244, 4.458)	0.009

Note: Model 1, unadjusted model. Model 2, model 1 + adjusted for age and sex. Model 3, model 2 + adjusted Charlson comorbidity index, primary renal disease, current smoking status, medication (ACEIs/ARBs, diuretics, number of anti-HTN drugs, statins), BMI, SBP and DBP. Model 4, model 3 + adjusted for hemoglobin, albumin, LDL-C, TG, fasting glucose, 25(OH) vitamin D, hs-CRP, eGFR, spot urine ACR, LVMI, and LVEF. Abbreviations: CKD, chronic kidney disease; CV, cardiovascular; CI, confidence interval; HDL-C, high-density lipoprotein cholesterol; HR, hazard ratio; ACEIs, angiotensin-converting enzyme inhibitors; ARBs, angiotensin receptor blockers; ; HTN, hypertension; BMI, body mass index; SBP, systolic blood pressure; DBP, diastolic blood pressure; LDL-C, low-density lipoprotein cholesterol; TG, total triglycerides; hs-CRP, high-sensitivity C-reactive protein; eGFR, estimated glomerular filtration rate; ACR, albumin-to-creatinine ratio; Q1, 1st quintile; Q2, 2nd quintile; Q3, 3rd quintile; Q4, 4th quintile; Q5, 5th quintile.

Table S3. HRs for the primary outcome by non-HDL-C level after excluding the subjects at CKD stage 5.

	Non-HDL-C	Events, *n* (%)	Model 1		Model 2		Model 3		Model 4	
			**HR (95%CIs)**	***P* value**	**HR (95%CIs)**	***P* value**	**HR (95%CIs)**	***P* value**	**HR (95%CIs)**	***P* value**
Composite CV event	Q1	85 (21.4)	2.327 (1.505, 3.597)	<0.001	1.968 (1.335, 2.901)	<0.001	1.877 (1.259, 2.800)	0.002	1.444 (0.838, 2.486)	0.186
	Q2	58 (14.4)	1.673 (1.060, 2.639)	0.027	1.640 (1.086, 2.478)	0.019	1.594 (1.050, 2.421)	0.029	1.563 (0.969, 2.521)	0.067
	Q3	37 (9.3)	Reference		Reference		Reference		Reference	
	Q4	50 (12.5)	1.362 (0.844, 2.196)	0.206	1.612 (1.053, 2.469)	0.028	1.497 (0.972, 2.304)	0.067	1.708 (1.020, 2.860)	0.042
	Q5	55 (13.2)	1.648 (1.037, 2.618)	0.034	1.643 (1.083, 2.493)	0.020	1.624 (1.062, 2.483)	0.025	2.274 (1.202, 4.300)	0.012

Note: Model 1, unadjusted model. Model 2, model 1 + adjusted for age and sex. Model 3, model 2 + adjusted Charlson comorbidity index, primary renal disease, current smoking status, medication (ACEIs/ARBs, diuretics, number of anti-HTN drugs, statins), BMI, SBP and DBP. Model 4, model 3 + adjusted for hemoglobin, albumin, LDL-C, TG, fasting glucose, 25(OH) vitamin D, hs-CRP, eGFR, spot urine ACR, LVMI, and LVEF. Abbreviations: CI, confidence interval; HDL-C, high-density lipoprotein cholesterol; HR, hazard ratio; Q1, 1st quintile; Q2, 2nd quintile; Q3, 3rd quintile; Q4, 4th quintile; Q5, 5th quintile.
